# Restrictive Transfusion Strategy Does Not Affect Clinical Prognosis in Patients with Ectopic Pregnancy

**DOI:** 10.1155/2017/2679148

**Published:** 2017-11-16

**Authors:** Yanjuan Huang, Yi Liang, He Ma, Mei Ling, Xuelian Ran, Jingxian Huang, Kejian Lu, Risheng Zhong, Fanke Huang, Wenwu Bin

**Affiliations:** ^1^Department of Anesthesiology, Third Affiliated Hospital, Guangxi Medical University, Nanning, Guangxi 530031, China; ^2^Department of Cardiology, Third Affiliated Hospital, Guangxi Medical University, Nanning, Guangxi 530031, China; ^3^Department of Gynecology, Third Affiliated Hospital, Guangxi Medical University, Nanning, Guangxi 530031, China

## Abstract

To assess the effects of restrictive transfusion strategy on hemoglobin (Hb) levels and prognosis in patients with ectopic pregnancy and severe hemorrhage undergoing emergency surgery, patient data were collected from 2012 to 2016. Following transfusion guidelines, restrictive transfusion was performed; at Hb levels of 60–70 to 100 g/L, transfusion was continued or not based on disease status. The patients were divided into four groups: blood loss < 400 ml (*N*1), 400–799 ml (*N*2), 800–1199 ml (*N*3), and ≥1200 ml (*N*4). Several prognosis parameters were assessed. Group* N*4 was further divided based on blood loss amounts (1200–1999, 2000–2999, 3000–3999, and 4000–5000 ml) for subgroup analyses. Blood loss, hemoglobin levels at discharge, and American Society of Anesthesiologists (ASA) scores were not associated with patient prognostic parameters, including intensive care unit (ICU) occupancy, cure, and healing rates, and surgical complications and hospital stay. No statistically significant difference was obtained in hospital stay among* N*1,* N*2, and* N*3 groups. Compared with* N*1 patients, cases with blood loss ≥ 1200 ml had significantly longer hospital stay. Interestingly, hospital stay was correlated with surgical approach, location of pregnancy, and operation time. Restrictive transfusion strategy could be safely used for emergency surgery in ectopic pregnancy with acute blood loss.

## 1. Introduction

Ectopic pregnancies occur at a rate of about 1-2% worldwide [1]. Current treatment protocols include the use of methotrexate; in hemodynamically unstable patients presenting acute abdomen pain or ectopic pregnancy rupture, the unique therapeutic option is surgery [2]. Transfusion therapy is usually required perioperatively in patients with ruptured ectopic pregnancy and severe blood loss [3, 4]. Guidelines for perioperative transfusion and adjuvant treatment proposed by the American Society of Anesthesiologists (ASA) in 2006 define the threshold value for erythrocyte infusion at hemoglobin (Hb) levels of 60 g/L, with no transfusion needed at Hb > 100 g/L; however, the approach for patients with Hb levels between 60 and 100 g/L was undefined [5]. The latest version of the American AABB transfusion guidelines of 2012 recommends a threshold for erythrocyte infusion of 70 g/L Hb in patients with stable disease [6]. Restrictive transfusion was proposed for transfusion standardization, reduction of transfusion cost, and surgical outcome improvement of cardiac operations [7]. However, several multicenter large sample studies [8–10] excluded patients with acute hemorrhage, active hemorrhage, and cardiovascular diseases, from their analyses. Transfusion practice varies considerably among surgeons and operation types, despite recommendations of restrictive use of blood products [11, 12]. Meanwhile, reports assessing restrictive transfusion strategy in ruptured ectopic pregnancy operation are scarce. Therefore, this study aimed to evaluate restrictive transfusion strategy, during emergency surgery, for its effects on Hb amounts and clinical prognosis in ectopic pregnancy with severe hemorrhage. Our findings demonstrated that restrictive transfusion strategy could be safely used for emergency surgery in patients with ectopic pregnancy and acute blood loss.

## 2. Materials and Methods

This retrospective cohort study assessed patients with ruptured ectopic pregnancy requiring emergency surgery in the Third Affiliated Hospital of Guangxi Medical University from January 2012 to May 2016. Restrictive transfusion strategy (Hb < 60–70 g/L was used as indicator of erythrocyte infusion; upon transfusion, Hb levels were maintained at 60–70 to 90 g/L) was used in emergency surgery for patients with ectopic pregnancy and severe blood loss. In July 2013, intraoperative autologous call backed transfusion was performed in patients with ectopic pregnancy.

The study protocol was approved by the Ethics Committees of the Third Affiliated Hospital, Guangxi Medical University, Nanning, and all participants provided written informed consent.

### 2.1. Diagnosis of Ectopic Pregnancy

Before operation, ectopic pregnancy was diagnosed based on disease history, blood extracted via colpocoeliotomia posterior, and B ultrasound and eventually confirmed during operation.

### 2.2. Inclusion and Exclusion Criteria

Patients who were diagnosed with ruptured ectopic pregnancy and received emergency surgery in our hospital were included in this study.

Exclusion criteria were as follows: (1) comorbidity of primary blood diseases; (2) ischemic heart disease; (3) severe hemorrhagic shock with cardiopulmonary resuscitation; (4) concomitant uterine and ectopic pregnancies; (5) combined hyperthyroidism requiring specific treatment; (6) use of anticoagulant drugs.

### 2.3. Grouping

In this study, average body weight was 50 kg, with an average blood volume of 4000 ml (50 kg × 8%). When acute blood loss was 20–40%, the patient was in a state of moderate shock; transfusion therapy can be considered with acute blood loss of 30% (1200 ml) [13]. Therefore, the patients were divided into four groups based on 10% blood volume loss (400 ml) increments: blood loss < 400 ml (*N*1), between 400 and 799 ml (*N*2), from 800 to 1199 ml (*N*3), and ≥1200 ml (*N*4). Group* N*4 was further divided based on blood loss amounts (1200–1999, 2000–2999, 3000–3999, and 4000–5000 ml) for subgroup analyses. Hb levels were compared at discharge between patients with autologous blood and those with nonautologous blood.

### 2.4. Anesthesia

Anesthetic drugs included midazolam, fentanyl, remifentanil, propofol, and cisatracurium. Perioperatively, limited fluid resuscitation was used, and the major crystalloid solution was sodium lactated Ringer's solution (Sichuan Kelun Pharmaceutical Co., Ltd., China). Hydroxyethyl starch 200/0.5 and sodium chloride injection (Hangzhou Minsheng Pharmaceutical Co., Ltd., China) were the main colloid solutions. In patients with blood pressure < 90/60 mmHg, appropriate vasopressors, such as ephedrine, dopamine, and norepinephrine, were used to maintain blood pressure at a normal level (Philips patients monitors, Germany, and Philips Medizin Systeme, Philips Medizin Systeme Boeblingen GmbH, Germany).

### 2.5. Surgical Approaches

Surgery was performed by attending gynecologists, while the surgical approaches were proposed by physicians in charge of each patient according to the disease state and patient choice. Laparoscope accounted for 98.8%, with 1.2% conducted because of patient wishes or because of critical condition requiring immediate surgery.

During the operation, some patients had concurrent diseases requiring surgery. Considering that the patients were of childbearing age, they were relatively fit in terms of health. To avoid injury during the second surgery, other operations were carried out in stable disease condition after consent of patients' family members. Some cases received myomectomy/ovarian cystectomy/tubal repair and orthopedic/enterolysis/bilateral tubal ligation/bilateral adnexectomy/teratoma cystectomy simultaneously.

### 2.6. Transfusion Strategy

The restrictive transfusion strategy was used in this study.

### 2.7. Indications of Allogeneic Erythrocyte Infusion

Based on guidelines for perioperative transfusion and adjuvant therapy published by the American Society of Anesthesiologists in 2006 and the American AABB transfusion guidelines of 2012, erythrocyte infusion was carried out with Hb level < 60–70 g/L and not needed if Hb level exceeded 100 g/L. For patients with Hb levels between 60–70 and 100 g/L, transfusion was performed or not according to the physicians, based on comprehensive factors of degree of anemia, cardiopulmonary decompensation function, metabolic rate, and age [5, 6].

### 2.8. Indications of Autologous Transfusion

Before the operation, blood loss was estimated to be 20% (above 800 ml) by the physicians; patients were advised to use autologous transfusion and agreed. For patients with Hb level between 60–70 and 100 g/L after autologous transfusion, allogeneic transfusion was considered, according to indications of allogeneic erythrocyte infusion.

### 2.9. Expected Effects of Transfusion

Elevated hemoglobin (Hb) and hematocrit (Hct) were obtained, with stable and normal blood pressure and heart rate; pulse oxygen saturation (SPO_2_) was 98–100%, and urine volume was normal. In addition, no adverse performances of cardiovascular compensatory function, such as dizziness, palpitations, chest distress, and chest pain, were found. If necessary, blood gas and acid-base balance were monitored. Electrolytes (potassium, sodium, chloride, and calcium ions) were normal.

### 2.10. Indications of Plasma and Cryoprecipitate Infusion

Coagulation function was monitored with standard laboratory diagnostic parameters, including blood platelet count, PT, APTT, INR, and fibrinogen. Plasma and cryoprecipitate were infused if PT or APTT was >1.5 times, or INR > 2.0, or the surgical wound showed blood infiltration, or fibrinogen concentration was less than 1.0 g/L.

### 2.11. Informed Consent for Transfusion

Before any transfusion therapy, the patients or their families were fully informed about the proposed plan, benefits to the patients, transfusion-related risks, and other alternative treatment options. The patients also provided signed informed consent. When a patient had indications for transfusion, blood was requested from the blood bank, and transfusion was performed.

### 2.12. Intraoperative Recovery Washing of Autologous Transfusion

A Beijing Jingjing autologous-P3000 blood recovery instrument (China) was used. The matching double-lumen suction line was employed to retrieve the hemoperitoneum; anticoagulation was performed per 100 ml of blood with 200 U heparin. At a certain amount, the recovered blood was submitted to centrifugation, washed, and pumped into blood recovery bags before transfusion to the patients. The washing solution was 0.9% sodium chloride injection, and usually 1000 ml solution was needed to wash 300 ml of erythrocyte suspension.

### 2.13. Observational Parameters

#### 2.13.1. Main Parameters

Hb levels at discharge, ICU occupancy rate, cure rate, wound healing grade, postoperative complications (postoperative infection and hemorrhage), and hospital stay were measured.

#### 2.13.2. Secondary Indicators

Operation time (from skin incision to surgical suture), anesthetic recovery time (from surgery end to anesthetic recovery and tracheal extubation), blood loss, number of allogeneic/autologous transfusions during hospitalization, transfusion rate, adverse reactions of transfusion (allergy, purpura, nonhemolytic febrile reaction, hemorrhage, hemolysis, transfusion-related acute lung injury), and anemia-related adverse reactions (dizziness, palpitation, chest distress, and chest pain) were assessed as well.

### 2.14. Blood Loss Assessment

Blood loss amounts included the sum of negative pressure suction bottle (with weight of washing solution subtracted), amount of autologous blood recovery, intraoperative blood blot, and cotton pads; blood volume of blood clots and cotton pads was calculated empirically.

Hb levels at discharge were measured at the Department of Medical Laboratory of our hospital, and blood cell composition was analyzed on a Sysmex xn-9000 (Japan) whole blood cell count instrument.

### 2.15. Treatment of Anemia

After transfusion therapy, most patients showed no anemia-related symptoms after the operation, and no other anemia-related therapy was conducted. For Group* N*4 patients with significant blood loss, especially when Hb levels were 56–80 g/L, anemia was corrected by the auxiliary method of intravenous iron supplementation for about 4 days.

### 2.16. Hospital Stay

Hospital stay after the operation was assessed for patients diagnosed with ectopic pregnancy that received preoperative conservative treatment.

### 2.17. Discharge Criteria

To be discharged, the patients had to have good general state, with stable and normal vital signs. Surgical incisions had to show class A healing. The patients had to show no wound pain and no anemia-related adverse reactions (dizziness, palpitation, chest distress, and chest pain). Under normal conditions, stitches can be removed five days after surgery. Patients with hospital stay below five days were required to return for stitch removal on the fifth day after the operation.

### 2.18. Cure Criteria

Cure was reflected by good general state, with stable and normal vital signs. Surgical incisions had to show class A healing. In addition, serum levels of human chorionic gonadotropin had to decrease to normal values.

### 2.19. Follow-Up after Discharge

The patients were required to have routine telephone follow-up after discharge by the hospital, with the department assigning a specific person for this purpose. In normal circumstances, records of written data were usually not needed. The cases received routine telephone follow-up within 2 weeks after discharge, and no operation-related and anemia-related complications were found. No patient needed to return for examination and treatment due to abnormal HCG results.

### 2.20. Statistical Analysis

The SPSS 22.0 statistical software was used for analysis. Count data were presented as median, range, and assessed by Kruskal-Wallis test. Categorical data were by chi-square test or Kruskal-Wallis test. *P* < 0.05 was considered statistically significant.

## 3. Results

### 3.1. Baseline Characteristics

During the study period, a total of 1140 cases were diagnosed with ectopic pregnancy and required emergency surgery in our hospital. Among them, the following 7 cases were excluded: 1 of concurrent primary thrombocytopenic purpura, 1 of leukopenia, 1 of ischemic heart disease, 1 with cardiopulmonary resuscitation, and surgery administered in the emergency department due to severe blood loss, 2 with concurrent uterine and ectopic pregnancies and 1 with combined hyperthyroidism. Finally, a total of 1133 cases were included in the final analysis. Baseline characteristics and perioperative data of the patients are summarized in Table 1. There were 550, 157, 201, and 225 subjects in Groups* N*1,* N*2,* N*3, and* N*4, respectively. Weights were 50 (38–88), 50 (39–85), 50 (30–85), and 50 (32–80) Kg in Groups* N*1,* N*2,* N*3, and* N*4, respectively (*P* = 0.018). Most patients were treated by laparoscopy; only 1 and 13 Groups* N*1 and* N*4 underwent laparotomy, respectively. There were significant differences in age, weight, surgical approaches, pregnancy location, and weeks of pregnancy.

### 3.2. Perioperative and Postoperative Findings

All patients were in stable condition after the operation, with no need for ICU treatment (one case had a blood loss of 5000 ml, but postoperative vital signs were stable; due to calendar problems, the patient was sent to the ICU department for observation). A cure rate of 100% was obtained. In addition, there were no surgical complications such as infection and wound hemorrhage (Table 2). Furthermore, no difference in hospital stay was observed among the groups, and clinical prognosis in various groups did not differ. Moreover, no transfusion-related adverse reactions (allergy, purpura, nonhemolytic febrile reaction, hemorrhage, and hemolysis) were noted, for both allogeneic and autologous transfusion cases. As shown in Table 2, ASA classes and operation and recovery times among the four groups were significantly different.

### 3.3. Hemoglobin Levels at Discharge

**Figure 1:**
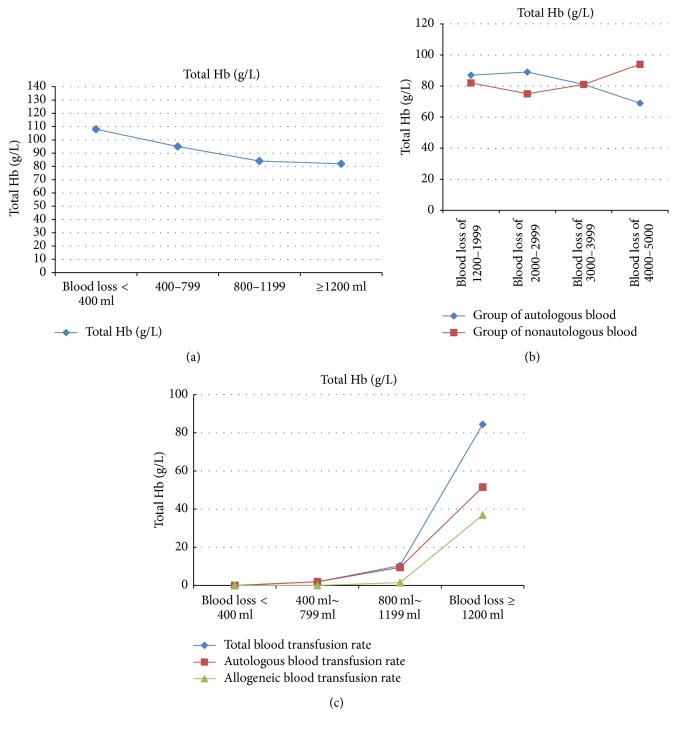
Perioperative transfusion rates and effect of blood loss on Hb level at discharge. The effect of blood loss on Hb level at discharge was assessed for the whole study population (a) and Group* N*4 subgroups (b). Perioperative transfusion rates (%) were assessed as well (c).

Total Hb decreased with increasing blood loss at discharge (Figure 1(a)). As for Hb levels, no clear trend was obtained for various subgroups of Group* N*4 in patients with autologous transfusion and those with nonautologous transfusion (Figure 1(b)).

### 3.4. Transfusion Rates

Autologous blood transfusion and blood transfusion rates were different among the four groups; meanwhile, no differences were found in allogenic blood transfusion (Table 3). Subgroup analysis of patients with blood loss ≥ 1200 ml (*N*4 group) was carried out. Interestingly, a statistically significant difference was found in Hb levels among various subgroups of patients with autologous blood transfusion (Table 4). Perioperative transfusion rates were generally lower for allogeneic blood transfusion than autologous transfusion (Figure 1(c)).

### 3.5. Associations of Various Clinical Parameters with Hospital Stay

As shown in Table 5, surgical approach (laparoscopy) and pregnancy location (abdominal cavity) were negatively correlated with hospital stay, while weight and operation time showed positive associations; the remaining parameters assessed showed no significant correlation, including age, pregnancy location, blood loss volume, and Hb at discharge. However, a poor fit was obtained in these multivariate analyses (*r*^2^ of 0.03).

## 4. Discussion

### 4.1. Surgical Transfusion Is a Double-Edged Sword

When the effective circulating blood volume reduces and blood composition changes, perioperative transfusion can correct anemia, enhance patient tolerance towards surgery, and promote postoperative wound healing as well as recovery [14]. However, Shander et al. [15] suggested that both transfusion and anemia are independent risk factors for organ damage and increased mortality. In addition, open transfusion does not reduce mortality in high-risk patients, for example, those with cardiovascular diseases, and could even increase adverse events such as postoperative infection [16] and impaired wound healing [17, 18] in critically ill individuals. Moreover, multiple studies have confirmed that transfusion has risks, including propagation of blood borne diseases [19, 20], allergic reactions [21], and transfusion-related acute lung injury [22, 23]. Discrepant supply and demand of medical use of blood have become increasingly evident, and shortage of blood resources is common [24].

### 4.2. Determination of Transfusion Threshold

Indications for transfusion in current guidelines were designed under a stable state, but patients with ectopic pregnancy and severe blood loss have acute illness, with short surgical time. These women have acute and active blood loss, and it is unclear under an emergency state, whether the body can tolerate transfusion due to pathological and physiological changes caused by massive blood loss. Interestingly, Gould et al. [25] demonstrated that for ICU patients with normal blood volume, similar or better effects are obtained compared with open transfusion, when hemoglobin threshold of the red blood cell transfusion is reduced to 70 g/L and maintained at 70–90 g/L. A study in 213 American hospitals found an Hb threshold of transfusion in ICU patients of 8.6 ± 1.7 g/dL [26]. A prospective study in Canada revealed an Hb threshold of transfusion in ICU patients of 73.5 ± 4.7 g/L, while 82 ± 6.5 g/L was obtained for patients with cardiovascular diseases [27]. Meanwhile, a retrospective study in Netherlands found Hb thresholds of transfusion for women with postpartum hemorrhage to be 64 g/L (without massive hemorrhage) and 81 g/L (massive hemorrhage) [28]. A recent Korean study demonstrated that transfusion should be avoided for obstetrics and gynecology patients of childbearing age, even those with severe anemia (Hb < 50 g/L). In this study, average hemoglobin levels in patients with anemia were 36.0 ± 8.9 g/L for the most affected cases, who did not receive transfusion; anemia was corrected by auxiliary methods such as iron supplementation and erythropoietin administration [29].

Clinically, we found that the health condition was generally good in patients with ectopic pregnancy, since they were of childbearing age. Meanwhile, compensatory ability of the body was good, and most studies assessing restrictive transfusion used 70 g/L as the threshold for transfusion. Therefore, according to international guidelines and findings of a large number of relevant studies, RBC infusion threshold was used for emergency surgery patients with ruptured ectopic pregnancy and severe blood loss; indeed, infusion of allogeneic erythrocytes can be considered with Hb below 70 g/L.

In this study, Hb levels at discharge in the group with the most severe blood loss (*N*4) was 82 (59–110) g/L, suggesting that maintaining lower hemoglobin levels in patients with ectopic pregnancy and acute severe blood loss can meet the requirements for body oxygen supply, consistent with the posttransfusion hemoglobin target of 70~90 g/L advocated by Hebert and Carson [30]. Subgroup analysis showed that Hb levels at discharge were similar among the four subgroups of the nonautologous blood group. However, statistically significant differences were found among the four subgroups of the autologous transfusion group; this might be because recovery blood was not proportional to blood loss, with large amounts of blood (more than 3000 ml) lost in a short time, with more blood clots, leading to less recovery blood volume.

### 4.3. Restrictive Transfusion Strategy Can Be Used for Critically Ill Patients

Controversy remains over the impact of restrictive transfusion strategy and open transfusion on clinical prognosis [31]. Multiple studies suggested that restrictive transfusion cannot only significantly reduce the number of transfusions but also significantly decrease the incidence rate of complications, while improving clinical prognosis [32, 33]. Restrictive transfusion strategy has also been widely used in critically ill patients. Holst et al. [34] demonstrated that prognosis of low (Hb < 70 g/L) and high (Hb < 90 g/L) thresholds of transfusion are similar in septic shock patients. Meanwhile, disease-free and overall survival rates of patients submitted to surgery for early non-small cell lung cancer and those infused with LPRC are lower than values obtained for patients not infused with LPRC [35]. However, in patients with upper gastrointestinal hemorrhage, restrictive transfusion could better improve prognosis than open transfusion [36]. By assessing 32449 patients who underwent coronary artery bypass surgery, Moskowitz et al. [37] found that compared with individuals administered regular transfusion, intraoperative transfusion and the incidence of postoperative complications are significantly reduced in patients tolerating anemia in the perioperative period (threshold of transfusion was 60~70 g/L Hb). Meanwhile, the mortality decreased significantly. Evaluating high-risk patients after hip fracture surgery, Carson et al. [10] found that open transfusion strategy is similar to restrictive transfusion, which does not reduce the mortality of elderly patients with high risk of cardiovascular or in-hospital mortality and cannot improve the ability of walking independently after 60 days.

We found that Hb levels at discharge were different among the four groups, but no difference was found in prognostic indicators of patients, including postoperative ICU occupancy rate, cure rate, rate of class A wound healing, and surgical complications (postoperative infection, hemorrhage).

Interestingly, there was no difference in hospital stay for patients with blood loss below 1200 ml. However, there was a slight difference between the* N*1 (blood loss < 400 ml) and* N*4 (blood loss ≥ 1200 ml). By multivariate analysis, hospital stay tended to be correlated with surgical approach, pregnancy location, and operation time. Laparotomy, abdominal pregnancy, and prolonged operation time can all affect hospital stay. However, hospital stay was not related to blood loss, hemoglobin at discharge, and ASA classification of the patients before the operation, suggesting that the restrictive transfusion strategy did not affect hospital stay of patients with ectopic pregnancy accompanied with acute severe blood loss.

### 4.4. Limitations

Blood volumes of blood clots and cotton pads were empirically evaluated in blood loss calculation, which may lead to inaccurate blood loss amounts. In addition, operation time and hospital stay are affected by the selection of surgical approaches (in the early stage of the study, laparoscope was used cautiously in patients with severe blood loss). Furthermore, simultaneously performed surgeries (in some cases, e.g., myomectomy/ovarian cystectomy/tubal repair and orthopedic/enterolysis/bilateral tubal ligation/bilateral adnexectomy/teratoma cystectomy) could influence the study findings. In the early stage of this study, the traditional concept that plasma infusion to patients with severe acute blood loss can improve prognosis was considered. Thus, indications of plasma infusion for nonautologous group patients were not strict, resulting in unnecessary transfusion and waste of blood, with increased risk of allogeneic transfusion. Finally, very low correlation coefficients were obtained in multivariate analyses, relativizing the associations described in this study.

## 5. Conclusions

Overall, this study demonstrated that the restrictive transfusion strategy can be used in emergency surgery patients with ectopic pregnancy and severe blood loss. In addition, Hb levels after transfusion were maintained at 60~80 g/L, which did not affect clinical prognosis. Restrictive transfusion strategy combined with autologous transfusion not only can save time but also possesses many advantages, such as reducing allogeneic transfusion and avoiding the risk of allogeneic transfusion.

## Figures and Tables

**Table 1:** Baseline data and perioperative data of the patients, which was revised on July 13th, 2016.

Parameters	Blood loss < 400 ml (*n*1 = 550)	Blood loss of 400–799 ml (*n*2 = 157)	Blood loss of 800–1199 ml (*n*3 = 201)	Blood loss ≥ 1200 ml (*n*4 = 225)	*P* value
Age (years)	30 (17, 45)	30 (17, 45)	28 (16, 44)	30 (17, 48)	0.031
Weight (Kg)	50 (38, 88)	50 (39, 85)	50 (30, 85)	50 (32, 80)	0.018
Surgical approaches					<0.001
laparotomy	1 (0.18%)	0 (0%)	0 (0%)	13 (5.78%)	
laparoscope	549 (99.82%)	157 (100%)	201 (0%)	212 (94.22%)	
Pregnancy location					0.004
oviduct	544 (98.91%)	156 (99.36%)	197 (98.01%)	217 (96.44%)	
Uterine horn	5 (0.91%)	1 (0.64%)	0 (0%)	7 (3.11%)	
Abdominal cavity	1 (0.18%)	0 (0%)	4 (1.99%)	1 (0.44%)	
Weeks of pregnancy (weeks)	7 (3, 13)	7 (3, 13)	7 (3, 13)	7 (4, 10)	<0.001

It can be seen from this table that there is significant difference among age, weight, surgical approaches, pregnancy location, and weeks of pregnancy.

**Table 2:** Operative data and prognosis parameters.

Parameters	Blood loss < 400 ml (*n*1 = 550)	Blood loss 400–799 ml (*n*2 = 157)	Blood loss 800–1199 ml (*n*3 = 201)	Blood loss ≥ 1200 ml (*n*4 = 225)	*P* value
ASA classification					<0.001
I	50 (9.09%)	9 (5.73%)	0 (0%)	0 (0%)	
II	496 (90.18%)	143 (91.08%)	193 (96.02%)	125 (55.56%)	
III	4 (0.73%)	5 (3.18%)	8 (3.98%)	89 (39.56%)	
IV	0 (0%)	0 (0%)	0 (0%)	11 (4.89%)	
Operation time	60 (20, 208)	65 (30, 235)	65 (30, 170)	80 (35, 222)	<0.001
Recovery time	5 (1, 30)	5 (1, 45)	8 (1, 25)	12 (2, 60)	<0.001
Hospitalization days	5 (2, 8)	5 (2, 14)	5 (3, 9)	5 (2, 9)	0.013
Cure rate	550 (100%)	157 (100%)	201 (100%)	225 (100%)	NA
ICU occupancy rate	0 (0%)	0 (0%)	0 (0%)	1 (0.44%)	0.519
Would healing classification					NA
Class A healing	550 (100%)	157 (100%)	201 (100%)	225 (100%)	
Class B healing	0 (0%)	0 (0%)	0 (0%)	0 (0%)	
Surgical complications					NA
Infection	0 (0%)	0 (0%)	0 (0%)	0 (0%)	
incision bleeding	0 (0%)	0 (0%)	0 (0%)	0 (0%)	
Adverse reactions of blood transfusion					NA
Allergy	0 (0%)	0 (0%)	0 (0%)	0 (0%)	
Purpura	0 (0%)	0 (0%)	0 (0%)	0 (0%)	
Nonhemolytic febrile reaction	0 (0%)	0 (0%)	0 (0%)	0 (0%)	
Hemorrhage	0 (0%)	0 (0%)	0 (0%)	0 (0%)	
Hemolysis	0 (0%)	0 (0%)	0 (0%)	0 (0%)	
Blood transfusion-related acute lung injury	0 (0%)	0 (0%)	0 (0%)	0 (0%)	
Anemia-related complications	0 (0%)	0 (0%)	0 (0%)	0 (0%)	NA
Dizziness	0 (0%)	0 (0%)	0 (0%)	0 (0%)	
Palpitation	0 (0%)	0 (0%)	0 (0%)	0 (0%)	
Chest distress, chest pain	0 (0%)	0 (0%)	0 (0%)	0 (0%)	
Hb level at discharge	108 (69, 136)	95 (70, 132)	84 (57, 102)	82 (57, 110)	<0.001

**Table 3:** Blood transfusion properties.

Parameter	Blood loss < 400 ml (*n*1 = 550)	Blood loss of 400–799 ml (*n*2 = 157)	Blood loss of 800–1199 ml (*n*3 = 201)	Blood loss ≥ 1200 ml (*n*4 = 225)	*P* value
Autologous blood transfusion (ml)	/	500 (500, 750)	775 (300, 1000)	1500 (750, 3000)	<0.001
Red blood cell (U)	/	/	3 (2, 4)	4 (1.5, 9)	0.645
Plasma (ml)	/	/	200	400 (150, 1000)	0.263
Blood transfusion rate	0 (0%)	3 (1.91%)	20 (9.95%)	190 (84.44%)	<0.001

**Table 4:** Subgroup analysis of patients with ≥1200 ml blood loss (*N*4 group) at discharge.

Parameters	Blood loss of 1200–1999 ml	Blood loss of 2000–2999 ml	Blood loss of 3000–3999 ml	Blood loss of 4000–5000 ml	*P* value
Cases with autologous blood transfusion (*n*)	50	57	7	2	
Hb level at discharge	86.5 (59, 109)	89 (58, 110)	81 (73, 93)	69 (66, 73)	0.045
Cases with non-autologous blood transfusion (*n*)	54	44	10	1	
Hb at discharge	82 (57, 96)	75.5 (62, 102)	81 (57, 101)	94	0.171

**Table 5:** Multivariate analysis of determining factors of hospital stay.

Factors	Standardized coefficient	*t* statistic	*P* value
Age	−0.053	−1.725	0.085
Weight	0.081	2.646	0.008
Surgical approach (laparoscope)	−0.073	−2.414	0.016
Pregnancy location (abdominal cavity)	−0.073	−2.475	0.013
Pregnancy location (cornual)	−0.003	−0.095	0.924
Operation time	0.077	2.429	0.015
Blood loss	0.049	1.276	0.202
Hb before discharge	0.013	0.352	0.725
